# Supplementary material for: Convergent innervations of mesencephalic trigeminal and vestibular nuclei neurons onto oculomotor and pre-oculomotor neurons—Tract tracing and triple labeling in rats
Source: PLoS One. 2022 Nov 28;17(11):e0278205. doi: 10.1371/journal.pone.0278205 (PMC9704657; doi:10.1371/journal.pone.0278205)
Supplement: Attachment — Submitted filename: Response to Reviewers.docx [file pone.0278205.s001.docx]

**Response to Reviewers**

*Reviewer #1*: The study by Chen et al., provides a morphological characterization showing the convergent projection from the mesencephalic trigeminal nucleus (Vme) and medial vestibular nucleus (MVN) onto motorneurons of the III and IV nuclei, as well as onto pre-motorneurons of the interstitial nucleus of Cajal (INC) and Darkschewitsch nucleus (DN).
The study in general is very well done. Combination of anterograde, retrograde and immnunostaining provides convincing experimental evidence supporting the conclusion that motorneurons and premotorneurons receive convergent innervation from the Vm and the MVN. This conclusion is supported by confocal microscopy showing presynaptic buttons from Vme and MVN neurons contacting the same cell body or dendrite, rising interesting perspectives on how these postsynaptic neurons integrate these inputs to control eye movements. Despite of that, some concerns arise.

*Answer:* we appreciate the reviewer’s valuable comment.

Major concerns:

*Reviewer:* Anterograde tracer (BDA) injected in the Vme yields a strong staining of the ipsilateral III and IV motor neuclei, as well as of the INC and DN. However, these injections yield only very weak staining of the trigeminal motor nucleus (Vmot), as shown in Figures 1F, 2A and 5D. This is surprising as Vme afferents (particularly spindle afferents) heavily project to the ipsilateral Vmot. This monosynaptic projection is very well documented in rodents, both electrophysiologically (Grimwood et al. 1992) and morphologically (Dessem et al. 1997; Luo and Li 1991; Luo et al. 1991; Stanek et al. 2014). Authors should provide an interpretation of this unexpected results of their experiments.

*Answer:* yes, the reviewer is correct, a large portion of Vme neuronal projections are going to Vmot. However, the projections from the Vme to the Vmot are actually collaterals of Vme neuronal central processes, based on results from intracellular horseradish peroxidase (HRP) labeling of masseter afferents by Dr. Shigenaga and colleagues [Shigenaga et al. 1988a, 1990]. The pseudo polar of single Vme neurons bifurcate to central and peripheral processes sooner or later away from the soma, the central axons consequently divide into a numerous collateral traveling to the Vmot, the supratrigeminal nucleus, the principle trigeminal sensory nucleus, the oralis of spinal trigeminal nucleus and the interstitial trigeminal regions and so on. Hence, the tract of tracer had been split into many different directions with a part of them reached the Vmot; so, the actual amount of tracer eventually arrived the Vmot may not be as much as we have expected.

On the other hand, the high molecular weight BDA travel slow through axons and degrade slow [Reiner et al. 2000]. We didn’t find published data about its degradation speed, but we believe it would degrade whenever they reached the destination, soma or terminal, like all other kinds of tracers those won’t stay there forever. The BDA would arrive the soma or bouton in the Vmot much earlier and degrade earlier. The distance between the Vme and Vmot is almost 1/6-8 of the distance between the caudal Vme and III based on a rat brain atlas [Paxinos and Weston 1998]. Thus, when the BDA densely filled in boutons at the III/IV and INC/DN, the tracer in the Vmot have been significantly degraded.

Aforementioned 2 reasons may help to interpret the phenomenon that the BDA labeling in the Vmot is much weaker comparing to that in the III/IV and INC/DN.

*Reviewer:* Results are discussed assuming that stained Vme neurons are only spindle afferents. However, the Vme also contain a large population of afferents innervating the periodontal ligament (Shigenaga et al. 1988). Moreover, the caudal portion of the Vme, where BDA injections were performed in this study by Chen and co-workers, contain a large proportion of these periodontal afferents. In that sense, most probably some of the anterogradely stained Vme afferents belong to this class. This possibility should be taken into consideration in the Discussion section.

*Answer:* right, the reviewer is correct. At the beginning, we discussed possible involvement of the periodontal afferent Vme neurons in this pathway. We cited a clinic entity of eyeball elevation by brushing teeth or squeezing gums [Gottlob et al., 2002], observed in a case of Marcus Gunn syndrome --- a congenital ptosis [Pratt et al., 1984; Brodsky CM, 2010]. However, we finally removed this part of discussion, as we were afraid that the readers would be confused, since our current study was not related to any neuro-ophthalmologic problem. While, as reviewer has indicated, the fact that the Vme, especially the caudal Vme, containing a large population of periodontal ligament afferent neurons [Shigenaga et al., 1988b; Nomura and Mizuno, 1985] could not be avoid. Therefore, we added a statement in Discussion (line 257- 260) as the follow: “However, the periodontal afferent Vme neurons could not be excluded since they are predominantly located in caudal part of the Vme [41], the area we injected the tracer. While, we considered jaw muscle afferent Vme neurons herein only because we had evidences that those neurons are involved in the related pathway [25-27].”

*Reviewer:* Minor concerns:

For consistency capitalize letters identifying panels in Figure 1.

Line 66: citation “2” should be between square brackets.

Lines 72-73: citations “16-19” should be between square brackets.

Line 120: citation “35” should be between square brackets.

Line 222: citation “35” should be between square brackets.

Line 249: should read “nucleus of posterior commissure”

Line 293: citation “46” should be between square brackets.

*Answer:* thank the reviewer very much for her/his time. Following the reviewer’s guide, we found some problems in our EndNote, so we have made efforts to fix the problem. This time we have carefully examined each line to avoid the same kind of mis-format.

Correspondingly, we have capitalized the letters identifying the panels in the Fig. 1.

References:

Shigenaga Y et al., Morphology of single mesencephalic trigeminal neurons innervating masseter muscle of the cat.

Brain Res 1988a, 445:392-399.

# Shigenaga Y et al., Two types of jaw-muscle spindle afferents in the cat as demonstrated by intra-axonal staining with HRP. Brain Res 1990, 514:219-237.

# Reiner A et al., Pathway tracing using biotinylated dextran amines. J Neurosci Method 2000, 103:23-37.

# Paxinos G and Weston C, The rat brain in stereotaxic coordinates. 1998, Springer, London, Boston, New York, Sydney, Tokyo, Toronto.

# Gottlob I et al., Elevation of one eye during tooth brushing. Am J Ophthalmol 2002, 134:459-460.

# Pratt SG et al., The Marcus Gunn phenomenon. A review of 71 cases. Ophthalmology 1984, 91:27-30.

Brodsky CM, Pediatric Neuro-Ophthalmology. Second ed. New York, Dordrecht, Heidelberg, London: Springer, 2010.

# Shigenaga Y, Yoshida A, Mitsuhiro Y, Doe K, Suemune S. Morphology of single mesencephalic trigeminal neurons innervating periodontal ligament of the cat. Brain Res 1988b, 448: 331–338.

Nomura S and Mizuno N, Differential distribution of cell bodies and central axons of mesencephalic trigeminal nucleus neurons supplying the jaw-closing muscles and periodontal tissue: a transganglionic tracer study in the cat. Brain Res 1985, 359:311-319.
