# Supplementary material for: Convergent innervations of mesencephalic trigeminal and vestibular nuclei neurons onto oculomotor and pre-oculomotor neurons—Tract tracing and triple labeling in rats
Source: PLoS One. 2022 Nov 28;17(11):e0278205. doi: 10.1371/journal.pone.0278205 (PMC9704657; doi:10.1371/journal.pone.0278205)
Supplement: Attachment — Submitted filename: Responses to Reviewers.docx [file pone.0278205.s002.docx]

Reviewer’s commend and our responses:

1. While I agree with the idea of a table in order to summarize data, the way Table 1 is constructed is confusing. It would be much valuable a summary of the different experiments with its corresponding successful rates, disregarding of the sex of the animals If this wasn’t systematically annotated. Include the number of experiment and the number of successful observations, for instance in how many experiments injecting BDA at Vme+PHA-L at MVN+Nissl did authors observed convergent innervation of III/IV neurons. Below is a posible organization for the table.

| Experiment | Observation | Total number of experiments |
| --- | --- | --- |
| BDA@Vme | axon terminals III/IV nuclei (n=x) |  |
| BDA@Vme+PHA-L@MVN+Nissl | convergent innervation of III/IV neurons (n=x) |  |
| BDA@Vme+PHA-L@MVN+ChAT immune | convergent innervation of III/IV motoneurons |  |
| BDA@Vme+PHA-L@MVN | INC |  |
| BDA@Vme+PHA-L@MVN+CTB@III | convergent innervation of INC neurons |  |

*Response:* We have revised the Table 1 based on the above format, and in the new Table 1, the success rate was listed since the previous review required to “describe the percentage of success in the total number of animals”. Please see new Table 1.

2. The criterion employed to consider a given injection as a successful one should be included in the text of the Materials and Methods section.

*Response:* Yeah, we have rephrased relative section of the Materials and Methods as you suggested. Please see the new-submitted manuscript in page 5, line 124 - 125 and line 131 - 132; and in page 7 line 165 - 166.

3. According to the author’s response to the second Reviewer, in a preliminary set of experiments the authors determined that injections in either right or left side, as well as in male or female animals, yield no difference and hence injections were done in either side and in animals of either sex. If this is the case, this should be stated in the Materials and Methods section.

*Response:* Yeah, we have added a new section in the Materials and Methods named as “***Sexual and side difference in BDA, PHA-L and CTB injections***”. Please see the new-submitted manuscript in page 7, from line 174 to 177.

4. The Figure 6 and associated discussion should be reformulated according to comments of the second Reviewer. While I agree that exists a large extent of similarity between rat’s neuronal circuit and human brain networks, the great difference of head posture between bipeds and quadrupeds, as recognized by the authors themselves, makes most of the conclusions highly speculative. Accordingly, in the Discussion section, extrapolation of findings in rat to explain neurological mechanism in human should be carefully done, and must include explicit considerations and limitations due to postural difference as mentioned above.

*Response:* We have added “a smaller rat diagram” in the Fig. 6 as suggested by the last reviewer. Correspondently, we diagrammatized location and extension direction of the rat’s jaw and neck muscles, and described the directions of different potential vectors during head rolling. Please see Fig. 6A in new-submitted manuscript, and the relevant discussion in page 12 line 285-286 and line 302-303.

**Recommended changes:**

1. Line 68 (resubmitted manuscript): The expression “VOR reflexive arc” is redundant. Also, clarify the whole sentence. A possible rephrasing: “Studies of VOR and its underlying mechanisms revealed that neuronal circuits executing horizontal…”

*Response:* Thank you for the suggestion. That has been amended (see page 3 line 58).

2. Line 75: Change to “participate in the perception of” or “sense”, “perceive” at it is stated imply a higher cognitive process carried by the individual.

*Response:* Yeah, we have changed this part. Please see the new-submitted manuscript page 3 line 65-66.

3. Line 100: “These facts infer that the pathway is possibly…” Please rephrase “suggest” or “indicate” instead of “infer”.

*Response:* Thanks. We have replaced “infer” with “suggest”. Please see the new-submitted manuscript page 4 line 87.

4. Line 106: “Ishii, the author, concluded that alternative change…” This expression is colloquial, please rephrase.

*Response:* Yeah, the sentence has been changed as the follow: “The author concluded that ∙∙∙ ∙∙∙ ∙∙∙” (Line 93).

5. “…,in addition to the VN efferent innervations onto the masseter motoneurons [21].” I think the use of plural is incorrect, check please.

*Response:* Thank you, amended (see new-submitted page 4 line 96).

6. Line 111: “reaching the oculomotor system may be interacted by aforementioned…” not clear, perhaps “integrated with” instead.

*Response:* Thank you for this direction, we have rephrased this sentence as you suggested. Please read new-submitted manuscript from line 97 to 100 in page 4.

7. Line 146: “, in the same parameter as aforementioned,” This refers to the ionophoretic injection parameters? If so, state it explicitly please.

*Response:* Yeah, this part was also rephrased as you suggested as the following: “2.5% PHA-L ··· ··· ···, was delivered with 2 Hz, 200 ms duration positive current output to the MVN ··· ··· ···” (see page 5, line128 – 129 of new-submitted).

8. Line 200: The previous conjugated is 568 and not 594, please check.

*Response:* Thank you and it was amended (see page 7, line 171 of new-submitted).
